# BRCA1 as a Therapeutic Target in Sporadic Epithelial Ovarian Cancer

**DOI:** 10.1155/2010/891059

**Published:** 2010-02-22

**Authors:** Katherine V. Clark-Knowles, Anna M. O'Brien, Johanne I. Weberpals

**Affiliations:** ^1^Centre for Cancer Therapeutics, Ottawa Hospital Research Institute, 501 Smyth Road, Ottawa, ON, Canada K1H 8L6; ^2^Division of Gynaecologic Oncology, The Ottawa Hospital, 501 Smyth Road, Ottawa, ON, Canada K1H 8L6

## Abstract

In sporadic epithelial ovarian cancer (EOC), the inactivation of BRCA1 through various mechanisms is a relatively common event. BRCA1 protein dysfunction results in the breakdown of various critical pathways in the cell, notably, the DNA damage response and repair pathway. Tumors from patients with *BRCA1* germline mutations have an increased sensitivity to DNA damaging chemotherapeutic agents, such as cisplatin, due to defective DNA repair. Thus, inhibiting BRCA1 in sporadic EOC using novel targeted therapies is an attractive strategy for the treatment of advanced or recurrent EOC. Several classes of small molecule inhibitors that affect BRCA1 have now been tested in preclinical and clinical studies suggesting that this is a rational therapeutic approach. The aim of this paper is to provide an understanding of how BRCA1 has evolved into a promising target for the treatment of sporadic disease and to outline the main potential small molecule inhibitors of BRCA1 in EOC.

## 1. Introduction

Up to 10% of epithelial ovarian cancers (EOCs) are caused by germline mutations in the tumor suppressor genes, *Breast Cancer 1* (*BRCA1)* and *BRCA2* [1, 2]. Carriers of these mutations have a risk of developing ovarian cancer of 18%–54% by age 70; rates significantly are higher than those of the general population [3]. The majority of sporadic EOCs display BRCA1 dysfunction or reduced expression, due to factors such as somatic mutations or promoter hypermethylation [4–6]. The *BRCA1* tumor suppressor gene codes for a 220 kD nuclear phosphoprotein which has been shown to be involved in many cellular processes such as cell cycle checkpoint control, DNA damage recognition and repair, apoptosis, the ubiquitin-proteasome pathway, and transcriptional regulation [7–10]. BRCA1 is located downstream in the cascade of the DNA damage sensors ATM and ATR and is phosphorylated by these kinases upon their activation in response to genotoxic stressors such as radiation and chemotherapeutic agents. Once in its phosphorylated state, BRCA1 becomes part of a number of different complexes which relocate to areas of damaged DNA and coordinate cell cycle checkpoints in order to execute DNA repair.

Ovarian cancer patients with tumors known to harbor a germline mutation in *BRCA1* are believed to display a better response to platinum-based therapies and improved survival compared to patients without *BRCA1*-associated disease [11]. BRCA1 deficiency is believed to result in deregulation of the carefully coordinated DNA repair cascade and thereby renders tumor cells more vulnerable to DNA damaging agents and genomic instability. While this may appear to be a distinct disadvantage for these cells in terms of tumorigenesis, this situation can be advantageous and potentially exploitable in the context of enhancing the response to DNA damaging chemotherapeutic drugs. The majority of patients demonstrate an initial response to debulking surgery along with the first-line therapy regimen of platinum and taxane-based agents. However, the majority will recur and develop platinum-resistance. To overcome platinum resistance, there is a significant need to develop novel therapeutic options that will either enhance the effectiveness of standard chemotherapeutics or target a subset of patient tumors based on molecular markers. This paper focuses on novel therapeutic drugs in sporadic EOC that directly or indirectly target BRCA1 and its interrelated pathways. A review of *BRCA1* gene therapy is provided as well as an overview of the preclinical and clinical studies on the most relevant small molecular inhibitors, poly(ADP-ribose) polymerase-1 (PARP), histone deacetylases (HDAC), checkpoint kinases (CHKs), and proteasome inhibitors in the context of how these agents alter the BRCA1 pathway to enhance sensitivity to platinum-based chemotherapy. Finally, the potential for clinical use of BRCA1 as a biomarker in EOC is reviewed.

## 2. Gene Therapy

The first efforts to target BRCA1 in EOC involved restoring BRCA1 function via gene therapy [12]. In its normal state, BRCA1 functions as a tumor suppressor gene, inhibiting the aberrant proliferation of tumor cells. However, BRCA1 rarely displays normal expression and function in EOC [5]. Thus, a logical therapeutic option is to restore the tumor suppressor function of BRCA1 in cancer cells in order to suppress cell proliferation. In a cell culture model, a normal splice variant of BRCA1 was overexpressed by a retroviral vector resulting in decreased cell proliferation. The cell line was then implanted into a mouse xenograft model and tumor growth suppression was observed [12]. Preclinical findings indicated that restoration of normal function of BRCA1, in a disease where its loss has been shown to contribute to both its development and progression, could have the therapeutic potential to inhibit tumor growth. In the Phase I trial, twelve patients with recurrent metastatic ovarian cancer, who had been treated with standard surgery and chemotherapy, received one to three cycles of intraperitoneal injections of BRCA1 in a retroviral vector. Two-thirds of patients demonstrated stable disease for 4–16 weeks and one third showed reduction of tumor burden. Given the absence of significant toxicity, a Phase II trial in patients with less advanced disease was performed [13]. This trial demonstrated little to no vector stability as well as a rapid development of a neutralizing antibody response, which was not observed in the previous trial. Furthermore, there was no evidence of clinical response. The authors postulated that this stark difference in results was likely due to differences in immunocompetence between the patient groups in each trial, attributable to differences in factors such as tumor burden, number of chemotherapy treatments, and nutritional status. The same group went on to design a second-generation retroviral vector containing *BRCA1*. In preclinical efficacy studies in mouse xenograft models, this new vector was found to be minimally immunogenic and increased survival compared to both the control vector and the first generation vector used in the Phase II trial [14]. No clinical trials with this vector have been reported to date. 

Vector reconstitution, in an attempt to regain normal function of BRCA1, has never proceeded to Phase III study. Thus, attention has recently focused on taking advantage of the inherent weakness of BRCA1-deficient tumor cells, namely, the inability to effectively repair DNA damage.

## 3. PARP Inhibitors

**Figure 1:**
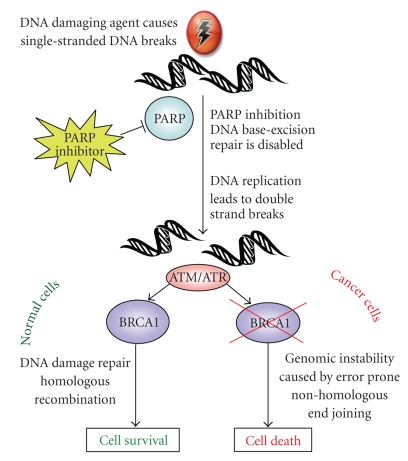
Targeting BRCA1 as a therapeutic strategy in the treatment of EOC. BRCA1 is central to the DNA damage response which is initiated following insults such as platinum-based chemotherapeutic agents. Various small molecule inhibitors may target BRCA1 directly or indirectly, ultimately leading to failure to repair damaged DNA and apoptosis.

A novel therapeutic option which has been the closest to widespread clinical use in the treatment of EOC is PARP inhibitors (PARPi) [15]. The PARP family of enzymes catalyzes the polymerization of poly ADP-ribose from a nicotinamide adenine dinucleotide (NAD^+^) substrate. They play a critical role in the repair of single-stranded DNA breaks (SSBs) via the base excision repair (BER) pathway and inhibition of PARP results in failure of SSB repair. Unrepaired SSBs which encounter a DNA replication fork result in double-strand breaks (DSBs). Normal cells are able to repair DSBs via the homologous recombination (HR) DNA repair pathway. However, cells with defective BRCA1 are unable to repair DSBs due to defective HR repair and are forced to use the error-prone nonhomologous end-joining (NHEJ) pathway. The ensuing genomic instability ultimately results in cell death (Figure 1). This ability to preferentially target BRCA1-defective cells and spare those with normal function made PARPi an attractive option for the treatment of ovarian and breast cancer patients with BRCA1 germline mutations. It is noteworthy that preclinical studies examining the effect of PARP inhibitors on BRCA-deficient cancers have focused on breast cancer models. 

The first preclinical work to demonstrate susceptibility to PARP inhibitor-induced cytotoxicity in *BRCA*-null cells was published simultaneously by two different groups in 2005 [16, 17]. Bryant et al. showed that *BRCA2*-null V-C8 cells were extremely sensitive to PARP1 inhibitors of varying potency and that this was likely due to their inability to execute effective HR repair. Farmer et al. also demonstrated similar findings in *BRCA2*−/− mouse embryonic stem (ES) cells, as well as *BRCA1*−/− ES cells. Treatment of V-C8 and *BRCA*-null ES cells with PARP inhibitors resulted in an increase in chromosomal instability, cell cycle arrest, and apoptosis. The results of the in vitro studies were validated in vivo by creating xenograft mouse models using the same V-C8 and *BRCA2*-null ES cell lines. It was consistently found that treatment of the mice with PARP inhibitors blocked the growth of *BRCA*-null tumors but had no significant effect on reconstituted *BRCA* control tumors.

PARPi have also been shown to enhance the cytotoxicity of platinum-based agents in vitro and in vivo, irrespective of *BRCA1* status. One study looked at a PARP-1 inhibitor, 3-aminobenzamide, and found increased cisplatin cytotoxicity in CH1cisR cisplatin-resistant ovarian tumor cells [18]. A novel 3-aminomethyl carbazole imide PARP-1 and PARP-2 inhibitor, CEP-6800, was combined with cisplatin to treat Calu-6-NSCLC cells [19]. Combination treatment displayed more DNA damage than cisplatin alone. Furthermore, when Calu-6-NSCLC tumor cells were implanted into a nude mouse model, there was a 35% reduction in tumor growth with CEP-6800/cisplatin combination treatment compared to single-agent cisplatin. 

There are preclinical data evaluating the combination of PARPi and platinum-based agents in both *BRCA*-mutant and BRCA-deficient breast cancer models. Donawho et al. used a *BRCA1*-deleted and a *BRCA2*-mutated MX-1 breast carcinoma xenograft mouse model to perform the in vivo evaluation of the Abbott Cancer Research PARPi, ABT-888. [20]. ABT-888 was shown to potentiate cisplatin and carboplatin cytotoxicity by inducing a greater regression of established tumors compared to modest tumor inhibition by platinum chemotherapy alone. Two studies analyzed a PARP-1 inhibitor by AstraZeneca (AZD2281) in BRCA-deficient models [21, 22]. In the study by Evers et al., AZD2281 displayed strong growth inhibition of BRCA2-deficient mouse mammary tumor cell lines compared to a BRCA2-proficient control tumor cell line [21]. Synergistic cytotoxicity in combination with cisplatin in the same model system was shown. Rottenberg and colleagues used the genetically engineered BRCA1-deficient breast cancer mouse model to establish AZD2281's efficacy alone and in combination with platinum-based agents [22]. When mice were treated with AZD2281 alone versus vehicle-treated controls, inhibited tumor growth and increased survival was observed. Subsequently, AZD2281 was combined with either cisplatin or carboplatin. This combination significantly prolonged the recurrence-free and overall survival compared to either platinum drug alone.

As a result of these promising findings, there are currently several PARPi in various stages of clinical development for use in patients with *BRCA1/2*-mutant breast and ovarian cancers (Table 1). The AstraZeneca/KuDOS compound KU-0059436 (AZD2281) was evaluated in *BRCA1/2* germline mutation positive breast, ovarian, and prostate cancers in a phase I trial [23]. This study showed that AZD2281 had few adverse effects, inhibited PARP and had antitumor activity in *BRCA1/2* mutation positive patients. 

A large proportion of sporadic EOCs display BRCA1 dysfunction and may behave similarly to *BRCA1* germline mutation-related disease in terms of overall survival, sensitivity to platinum drugs and defects in DNA damage repair. Given these findings, it is rational to extrapolate the use of PARPi for the treatment of patients with sporadic EOC. A number of clinical trials are currently underway examining the effect of PARPi alone or in combination with platinum agents in ovarian cancers irrespective of *BRCA1/2* mutation status (Table 2).

## 4. HDAC Inhibitors

**Figure 2:**
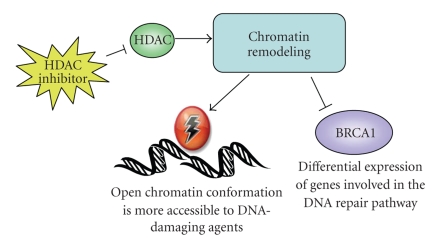
HDAC inhibition induces DNA damage and disrupts DNA repair. Inhibiting HDAC causes hyperacetylation of DNA and chromatin remodeling leading to more relaxed and open DNA. This conformational change renders DNA more accessible to cytotoxic DNA-damaging agents causing both upregulation and downregulation of genes in the DNA damage and repair cascade.

Histone Deacetylase Inhibitors (HDACi) have recently generated interest as a potential therapeutic option in the treatment of cancer, having demonstrated their ability to inhibit the proliferation of cancer cell lines in vitro and in vivo [31]. Histone deacetylases are enzymes involved in the posttranslational regulation of chromatin structure [32]. Their role is to catalyze the removal of acetyl groups from lysine residues in the core histones of chromatin, resulting in a more compact and transcriptionally repressed chromatin structure. The mechanism by which HDACi suppress the growth of cancer cells might be due to their inhibition of acetyl group removal, resulting in the hyperacetylation of chromatin structure. This causes chromatin to become more relaxed and open, making it more accessible to DNA-damaging agents and changing the expression of genes in the DNA damage recognition and repair cascade (Figure 2). There are several classes of HDACi including hydroxamic acid-derived compounds (e.g., Trichostatin A and SAHA), short-chain fatty acids (e.g., Sodium butyrate and valproic acid), benzamides, cyclic peptides, and thiolates.

HDACi may have a significant effect on the sensitivity of EOC tumors to DNA-damaging agents. Zhang et al. performed a gene expression profile on squamous carcinoma cells and observed that most genes involved in cell cycle control, DNA replication, and DNA damage repair were downregulated when treated with Trichostatin A (TSA) [33]. Our group has shown that the treatment of A2780s/cp ovarian cancer cells with the TSA analogue, M344, causes the downregulation of BRCA1 mRNA and protein levels [34]. We have also shown that M344 was able to increase the sensitivity of A2780s/cp ovarian cancer cell lines to cisplatin and carboplatin, but not to taxol. Strait and colleagues showed that TSA alone induced apoptosis in cisplatin resistant ovarian cancer cell lines OVCA-3 and SKOV-3 [35]. Another study looked at several different HDACi and found that they all enhanced the cytotoxicity of cisplatin, but not to metabolic antagonists or microtubule-damaging agents, in six human ovarian cancer cell lines of varying cisplatin sensitivity [36]. R306465 and PXD101, hydroxamate-based HDACi, have shown efficacy in A2780 xenograft mouse models [37, 38]. Oral administration of R306465 in immunodeficient mice was well tolerated and antitumor activity of 76%–87% was observed compared to vehicle controls. PXD101 showed single-agent antitumor effect in xenograft mice that was enhanced by the combination with carboplatin treatment.

SAHA (vorinostat) has been approved for the treatment of cutaneous T-cell lymphoma and subsequently, a number of clinical trials are currently underway to evaluate the toxicity and dose of HDACi in solid tumors, including ovarian cancer (Tables 1 and 2). A phase II study of SAHA in recurrent ovarian cancer found that the treatment was well tolerated but had minimal activity as a single agent [24]. There are phase I/II trials underway examining the combination of taxol, carboplatin, and SAHA as well as carboplatin, gemcitabine, and SAHA. A phase III trial is recruiting advanced ovarian cancer patients for treatment with magnesium valproate, an HDACi, in combination with hydralazine, an antihypertension agent. Since current trials have focused on all ovarian cancer patients with advanced/recurrent disease, there may also be a future role for targeting a specific subset of the patient population based on tumor biomarkers.

## 5. CHK1/2 Inhibitors

**Figure 3:**
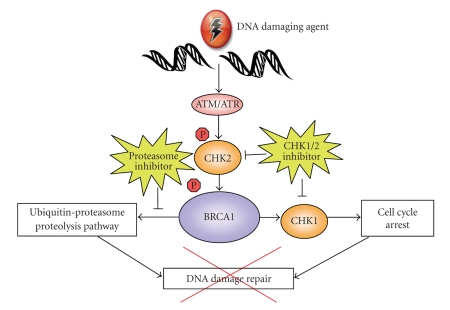
CHK1/2 and proteasome inhibition cause defects in the DNA damage repair pathway. CHK1 and CHK2 function to arrest the cell cycle when DNA has been damaged, thus allowing time to repair the DNA lesions. CHK inhibition leads to continued cell cycling in the presence of damaged DNA and is thought to alter the function of the DNA repair pathway.

Following DNA damage, BRCA1 is involved in the control of cell cycle checkpoints, which represents another potential mechanism to target BRCA1 therapeutically. Two genes involved in this aspect of the DNA damage cascade are Checkpoint Kinase 1 and 2 (CHK1 and CHK2). CHK1 is activated by ATR in response to stressors such as replication stress, chemotherapeutic agents, and SSBs; whereas CHK2 is activated by ATM in response to ionizing radiation, chemotherapeutics, or DSBs [39]. Activation of CHK1/2 leads to arrest of the cell cycle at different phases depending on the specific kinase activated, allowing for DNA repair to occur. The functional BRCA1 protein has been shown to be phosphorylated and activated by CHK2, resulting in the activation of CHK1. The presence of phosphorylated BRCA1 affects the expression and localization of Cdc25C, a downstream target of CHK1 [40]. Inhibitors of CHK1/2 abrogate normal cell cycle arrest induced by their activation, thereby preventing the repair of DNA damage (Figure 3). Altering the function of the checkpoint kinases may directly or indirectly impact BRCA1 function and thus may be a suitable target for therapy in EOC.

Husain et al. found that the CHK inhibitor UCN-01, a staurosporine derivative, potentiated the cytotoxicity of cisplatin in a panel of ovarian cancer cells, with a notable increase in apoptosis [41]. Furthermore, the cytotoxic effect was more pronounced in *p53*-wildtype cells. A phase I clinical trial of UCN-01 in combination with topotecan was performed in patients with advanced solid tumors, including a significant proportion of EOC [25]. This treatment combination demonstrated some efficacy and overall was well tolerated. However in the Phase II trial examining the same treatment in patients with advanced recurrent ovarian cancer, no significant antitumor effect was seen [26]. The efficacy of CHK inhibitors in the context of BRCA1 expression levels in EOC has not been examined, but warrants investigation due to the interaction between CHK1/2 and BRCA1 in the DNA damage cascade.

## 6. Proteasome Inhibitors

*BRCA1* is known to have a role in the ubiquitin-proteasome proteolysis pathway, whereby damaged and misfolded proteins are tagged with a polyubiquitin chain and targeted for ATP-dependent degradation by the 26S proteasome [42]. *BRCA1* contains a zinc ring finger domain in its amino-terminal region which has E3 ubiquitin ligase activity and aids in the transfer of ubiquitin to the target substrate. Mutations in the RING finger domain of *BRCA1* are thought to predispose to the development of cancer because they abrogate ubiquitin ligase activity [43]. It has been suggested that this particular function of BRCA1 may be critical to the DNA recognition and repair process. As such, inhibitors of the ubiquitin-proteasome pathway may offer an alternative therapeutic option in EOC, as inhibition of this pathway may result in defective repair of DNA damage (Figure 3). 

Proteasome inhibitors include compounds such as peptide aldehydes, boronates, and epoxyketones as well as *β*-actones, which prevent the degradation of ubiquitinated proteins. Several groups have demonstrated that the treatment of ovarian cancer cell lines with a proteasome inhibitor in combination with cisplatin treatment increased the cytotoxicity of platinum drugs, increased DNA damage and inhibited repair. Mimnaugh et al. pretreated ovarian cancer cells with either ALLnL or lactacystin proteosome inhibitors prior to cisplatin treatment and observed an abrogation in the expected increase in excision repair cross-complementation group 1 (ERCC1) expression with cisplatin and more efficient apoptosis [44]. The same group also evaluated the combination treatment of the proteasome inhibitor lactacystin with cisplatin in cisplatin-resistant ovarian cancer cells [45]. They observed the suppression of ERCC1 expression and inhibition of DNA repair with resultant enhanced cisplatin cytotoxicity. In addition, the proteasome inhibitor ALLnL was used in combination with cisplatin treatment in A2780s and A2780cp ovarian cancer cells, a cisplatin sensitive/resistant pair, and OVCAR3 cells [46]. They again found that cisplatin sensitivity was increased in all of the cell lines, along with an increase in DNA damage and defect in repair. They also noted a significant increase in the accumulation of cisplatin in the cells and a reduction in cisplatin efflux. Recent work by Jandial et al. has shown that the reduction of cisplatin efflux in ovarian cancer cotreated with the proteasome inhibitor, PS-341, is due to the prevention of cisplatin-induced downregulation of the copper transporter 1 (CTR1), a major transporter of platinum drugs [47].

Based on the preclinical results, proteasome inhibitors, namely, PS-341, have entered into clinical trials, including studies in EOC. In a Phase I trial examining a combination of carboplatin and PS-341 in recurrent EOC, an overall response rate of 47% was observed, including two patients demonstrating a complete clinical response, one of whom had platinum resistant disease [27]. Another recent Phase I trial evaluated PS-341 in combination with carboplatin in ovarian cancer patients with recurrent and platinum/taxane-resistant disease [28]. The drug combination was well tolerated, with just under half of the patients demonstrating stable disease. There are currently Phase II clinical trials assessing PS-341 either as a single agent or in combination with other therapies in ovarian cancer presently underway (Tables 1 and 2) [29, 30]. The combination of proteasome inhibitors to platinum chemotherapy taking account BRCA1 mutation status or expression levels is a potential area of future study in EOC.

## 7. BRCA1 as a Biomarker in EOC

A range of preclinical studies using both in vitro and in vivo models have supported the association of low BRCA1 mRNA and protein expression with an enhanced sensitivity to platinum agents [48, 49]. An ovarian cancer cell line SKOV3, which expresses high levels of BRCA1, was the model used to assess the role of BRCA1 in cisplatin sensitivity. Husain et al. depleted BRCA1 levels by an antisense inhibition approach to sensitize SKOV3 cells to cipslatin [48]. Zhou et al. used a retrovirus-mediated siRNA interference approach to show similar results [50]. Another group used both BG-1 and OVCAR5 ovarian cancer cell lines that were either stably transfected with a BRCA1 antisense construct or transiently transfected with a siRNA against BRCA1 [49]. The cells that displayed reduced BRCA1 expression were more sensitive to platinum agents than their empty vector and scrambled oligonucleotide controls. Using two different models to target the inactivation of Brca1 in mouse ovarian surface epithelial cells, an increase in chemosensitivity and an enhanced apoptotic response to cisplatin in the absence of Brca1 in this tissue was reported, suggesting that this phenomenon is also present in normal cells [51, 52]. Furthermore, in the ID8 mouse EOC cell line, Brca1 expression has also been shown to mediate sensitivity to platinum agents [53].

EOC patients with germline mutations in the *BRCA1* tumor suppressor gene have an improved initial response to treatment with platinum-based chemotherapy regimens and have an improved overall survival [54]. Several studies have also shown a reduction in BRCA1 expression in sporadic EOCs compared to normal ovarian tissue, as assessed by methods such as immunohistochemistry (IHC), loss of heterozygosity, mRNA levels, and hypermethylation of the *BRCA1* promoter [55]. Recent data also indicates that BRCA1 levels may be predictive of response to treatment and overall survival in sporadic EOC. In the largest study of 230 patients with sporadic EOC, Thrall et al. analyzed BRCA1 protein expression by IHC and found that decreased BRCA1 expression was protective for survival [56]. IHC was performed on formalin-fixed-paraffin-embedded (FFPE) samples with the mouse monoclonal antibody specific to the amino-terminal of the BRCA1 protein. The study was scored based on previously published methods used in breast cancer [57], with a score of 0–4, based on the number of cells stained in a field of view. Several other groups have also assessed BRCA1 protein levels via IHC in sporadic EOC in smaller sample sizes, with reduced expression found in between 34% and 90% of tumors [58–60]. The studies by Wang et al. and Zheng et al. followed the same experimental conditions as Thrall's group in terms of using FFPE samples and the same BRCA1 antibody epitope. Russell's study performed IHC on flash frozen sections and they used six different antibodies ranging in epitope from the amino to the carboxy terminus. With this rigorous approach, they were able to find reduced or absent BRCA1 protein expression in 90% of their cases. However, it must be considered that such a wide range of results may be attributed to variations in inclusion criteria as well as the relatively subjective nature of IHC scoring.

The group of Quinn et al. was the first to report that decreased BRCA1 mRNA expression by quantitative RT-PCR in tumors from patients with sporadic EOC who received platinum-based chemotherapy was predictive of an improved overall survival [49]. Our recent study substantiates these results by showing that lower BRCA1 expression predicts for longer overall survival, especially in patients who were optimally debulked <2 cm at the time of staging laparotomy [34]. While RNA analysis is a more quantitative approach, it not only requires the availability of frozen tissue, but RNA extraction is a more time-consuming approach than IHC on samples which are processed on a tissue microarray. Furthermore, unless the mRNA analysis has been done from microdissected samples, the tissue sample itself may be a mixture of heterogeneous tissue including normal, nonmalignant tissue.

A consistent finding in the few studies on human EOC tumors is that BRCA1 mRNA and protein are frequently expressed at low levels within cell nuclei relative to the commonly used positive tissue control MCF7, a breast cancer cell line. This may represent a potential challenge in utilizing BRCA1 as a clinically useful predictive marker. Distinguishing “high” expressors from “low” expressors can be particularly difficult, especially at the protein level via IHC, where the scoring method is usually qualitative. Quantitative methods such as analysis of mRNA levels may provide more accurate results and could facilitate differentiating between true high and low expressors in a population where baseline levels are low. Considerable success using this method has been achieved in the use of BRCA1 as a predictive marker in nonsmall cell lung cancer (NSCLC) [61].

## 8. Conclusion

The array of cellular processes in which BRCA1 plays an integral role offers several mechanisms by which its function could be targeted for the treatment of EOC. All of these options take advantage of the weakness that is central in a BRCA1-deficient cell, the inability to effectively repair damaged DNA. As a result, the therapies outlined in this review offer promise not just in *BRCA1* mutation-associated EOC, but to the large proportion of patients with sporadic disease with tumors that display BRCA1 deficiency due to epigenetic changes. Furthermore, as BRCA1 shows promise as a prognostic and predictive marker in sporadic EOC, patients identified as being high expressors could be treated with agents that downregulate BRCA1, thus sensitizing them to standard therapies. Further work, both in vitro and in clinical trials, is needed to assess the correlation between BRCA1 expression levels and response to these potential targeted therapies.

## Figures and Tables

**Table 1:** Completed clinical trials evaluating potential drug targets of BRCA1.

Drug class	Compound	Phase	Study population
PARP inhibitors	KU-0059436 (AZD2281)	Phase I	BRCA1/2 germline mutation in advanced breast and EOC [23]

HDAC inhibitors	SAHA (vorinostat)	Phase II	Recurrent EOC [24]

CHK inhibitors	UCN-01 (staurosporine derivative) in combination with topotecan	Phase I	Advanced solid tumors, including EOC [25]
	UCN-01 in combination with topotecan	Phase II	Recurrent EOC [26]

Proteasome inhibitors	PS-341 (bortezomib) in combination with carboplatin	Phase I	Recurrent EOC [27]
	PS-341 in combination with carboplatin	Phase I	Platinum/taxane resistant EOC [28]
	PS-341	Phase II	Recurrent, platinum-sensitive EOC [29]
	PS-341 in combination with paclitaxel	Phase I	Advanced solid tumours, including EOC [30]

**Table 2:** Ongoing clinical trials evaluating potential drug targets of BRCA1.

Drug class	Compound	Phase	Clinical trial number	Study population
PARP inhibitors	MK4827	Phase I	NCT00749502	EOC
	AG014699	Phase II	NCT00664781	Advanced EOC
	KU-0059436 (AZD2281)	Phase I	NCT00647062	EOC with or without BRCA1 mutation
	KU-0059436 (AZD2281) in combination with doxorubicin	Phase II	NCT00628251	BRCA1/2 mutation positive EOC
	ABT-888 in combination with bevacizumab, carboplatin, paclitaxel	Phase I	NCT00989651	EOC
	ABT-888 in combination with temozolomide	Phase I	NCT00526617	EOC
	KU-0059436 (AZD2281)	Phase II	NCT00679783	EOC with or without BRCA1 mutation
	KU-0059436 (AZD2281)	Phase II	NCT00753545	Platinum sensitive serous EOC
	BSI-201	Phase II	NCT00677079	Advanced EOC
	ABT-888 in combination with topotecan	Phase I/II	NCT01012817	EOC

HDAC inhibitors	SAHA (vorinostat) in combination with paclitaxel, carboplatin	Phase I/II	NCT00772798	EOC
	SAHA (vorinostat) in combination with carboplatin, gemcitabine	Phase I/II	NCT00910000	EOC
	Hydralazine and magnesium valproate	Phase III	NCT00533299	Advanced EOC

CHK inhibitors	UCN-01 in combination with irinotecan	Phase I	NCT00031681	Metastatic EOC

Proteasome inhibitors	PS-341(bortezomib) in combination vandetanib	Phase I/II	NCT00923247	EOC
	PS-341	Phase II	NCT00023712	Platinum-sensitive EOC
